# Recurrent dislocation of binocular crystal lenses in a patient with cystathionine beta-synthase deficiency

**DOI:** 10.1186/s12886-021-01974-8

**Published:** 2021-05-13

**Authors:** Ning Hua, Yuxian Ning, Hui Zheng, Ledong Zhao, Xuehan Qian, Charles Wormington, Jingyun Wang

**Affiliations:** 1 Tianjin Key Laboratory of Retinal Functions and Diseases, Tianjin Branch of National Clinical Research Center for Ocular Disease, Eye Institute and School of Optometry, Tianjin Medical University Eye Hospital, No. 251 Fukang Road, Nankai District, Tianjin, China; 2 Tianjin Eye Hospital, Tianjin, China; 3 Salus Univerisity Pennsylvania College of Optometry, Elkins Park, PA USA; 4 SUNY College of Optometry, New York, NY USA

**Keywords:** Case report, Cystathionine beta-synthase (CBS), Homocystinuria, Lens ectopia

## Abstract

**Background:**

Ectopia lentis is the common ocular manifestation of homocystinuria resulting from cystathionine beta-synthase (CBS) deficiency which has a high risk of thromboembolic complications.

**Case presentation:**

The present study reports the case of a teenager with recurrent lens dislocation and glaucoma. He was diagnosed with CBS deficiency according to a high level of serum homocysteine and compound heterozygous mutations at two different positions on the CBS gene. Antiglaucoma eyedrops and a mydriatic successfully controlled the intraocular pressure, while oral pyridoxine and betaine uptake lowered the serum homocysteine level effectively.

**Conclusions:**

Children with CBS deficiency may suffer from ectopia lentis, glaucoma and/or amblyopia. We firstly discovered a new mutation of CBS c. 697 T > G which had not been reported before. The patient was pyridoxine responsive and well controlled by medicine.

## Background

Homocystinuria due to cystathionine beta-synthase (CBS) deficiency, also known as classical homocystinuria (OMIM.org #236200), is a rare recessive inherited amino acid metabolism disorder that involves the pathway for cystathionine synthesis. Patients with CBS deficiency manifest a variety of disorders involving multi-systems occurring from neonates to adults. The infantile presentation is the most clinically severe, and includes hypotonia, seizures, apneas and/or coma [1, 2]. Adolescents or adults may present with intellectual disability/developmental delay, osteoporosis, psychiatric manifestations, and ‘marfanoid’ habitus [1, 3, 4]. Ocular manifestation mainly includes crystalline lens dislocation, which might be misdiagnosed as Marfan’s syndrome. However, CBS deficiency is highly correlated with thrombosis, which is the main cause of mortality [3]. The proper diagnosis is vital for clinicians to provide reasonable management.

We herein report a case of a 14-year-old boy with CBS deficiency presenting with recurrent dislocation of the crystalline lens from the anterior chamber to the vitreous cavity. In addition, we found compound heterozygous mutations at two different positions on the CBS gene. One of the genetic variants, c.697 T > G (p. W233N), has not been reported before.

## Case presentation

A 14-year-old boy was referred to the Department of Pediatric Ophthalmology and Strabismus at Tianjin Medical University Eye Hospital due to conjunctiva injection at the left eye with blurring for nearly 1 month. He had severe myopia with poorly corrected vision at a very young age. Since being a toddler, he was always the tallest in classes. However, the parents complained about their son’s bad temper and academic retardation. Seizures or thrombosis were denied. The boy had a ‘marfanoid’ habitus with scoliosis and arachnodactyly.

**Fig. 1:**
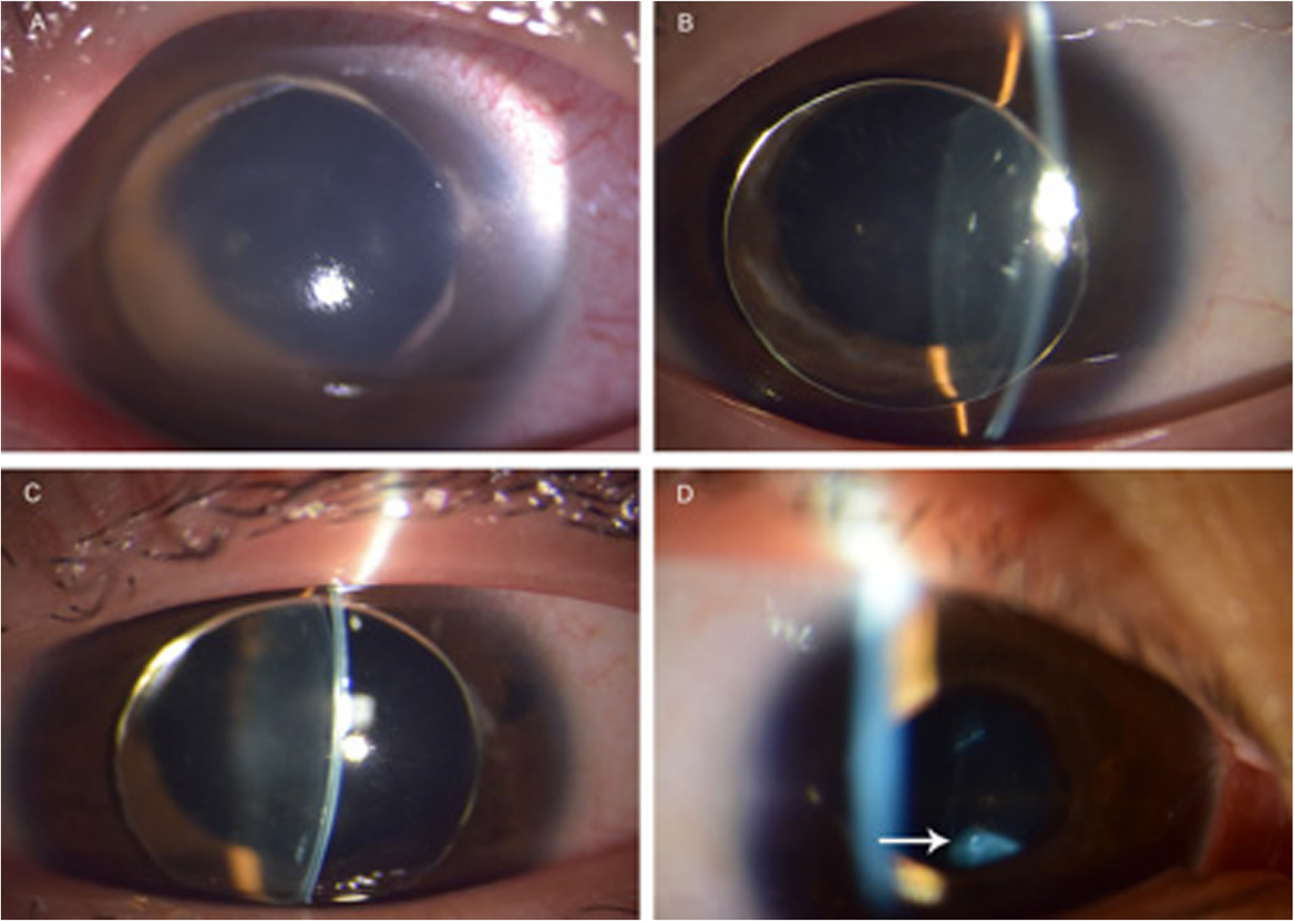
The changes of the lens location during medical control of intraocular pressure. **a**. The crystalline lens was totally dislocated into the anterior chamber in the left eye with corneal edema due to pupillary-block glaucoma; the intraocular pressure was 45 mmHg. **b**. The same lens dislocated in the right eye with normal intraocular pressure of 16 mmHg; the iris atrophy was clearly observed around the pupil. **c**. With control of the pressure, the corneal edema disappeared in the left eye and the lens was still in the anterior chamber. Marginal atrophy around the pupil was apparent. **d**. After the patient stood up from lying on his back, the spontaneous backwards movement of lens into the vitreous cavity in the left eye was detected. The white arrow indicated the dislocated lens in the inferior vitreous cavity

The uncorrected visual acuity was 6/120 in the right eye and finger counting in the left eye. The crystalline lenses were located in the anterior chamber binocularly, while the intraocular pressure was 45 mmHg in the left eye with conjunctiva injection (Fig. 1a), and 16 mmHg in the right eye (Fig. 1b). Simultaneously, bilateral iris atrophy around the pupil was detected.

The initial management included anti-glaucoma eye drops, carteolol hydrochloride 2% and brinzolamide 1% for daily use, and oral methazolamide 25 mg Q8H. The intraocular pressure became normal with the disappearance of corneal edema in the left eye after 2 days of treatment. (Fig. 1c).

In addition, the crystalline lens in the right eye was found to be in the inferior vitreous cavity (Fig. 1d) with a strand of elongated zonular fibers at 12 o’clock to the ciliary body. The refractive error was + 12.00/− 2.00 × 80 (SE + 11.00D) in the right eye with the lens in the inferior vitreous, and -18.00/− 4.00 × 30 (SE − 20.00D) in the left eye with the lens in the anterior chamber. The best-corrected visual acuity was 6/12 in the right eye and 6/30 in the left eye.

Since then, careful slit lamp examination showed the recurrent changes of the optic lens from the vitreous cavity to the anterior chamber during 5 days of observation. There were several lens position changes in a single day, accompanied with a posture change from the supine position.

**Fig. 2:**
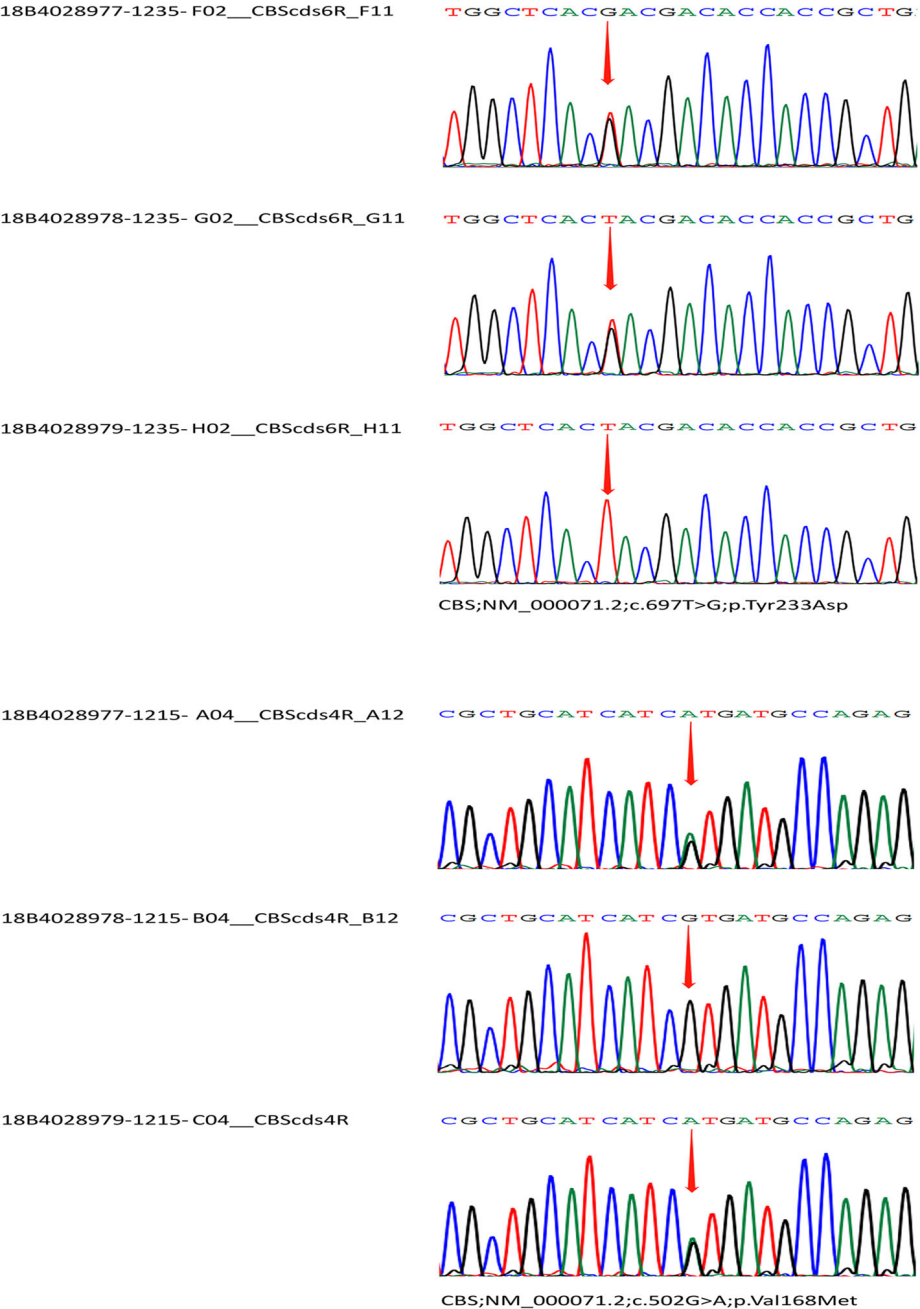
Sanger sequencing of the patient and the parents. Sample 18B4028977 was from the patient, 18B4028978 was from the mother and 18B4028979 was from the father

The plasma homocysteine concentration was 309.6 μmol/L, over 20 times the normal laboratory level (under 15 μmol/L). Whole exome sequencing and Sanger sequencing for the genetic variants (GBI Shenzhen, China) was performed from the blood samples of the patient and his parents. The result showed gene mutations in the CBS gene at two different positions, NM_000071.2(CBS): c. 502G > A, and NM_000071.2(CBS): c.697 T > G (Table 1). The mutation c.502G > A was inherited from his father, who was heterozygous, while the mutation c.697 T > G was inherited from the heterozygous mother (Fig. 2). Although the parents had different mutations in the two positions of the CBS gene, both were normal not only in general systems but also in eyes with normal vision.

**Table 1:** Gene detections of the patient and his parents

Gene detection	Gene	Exon	Genome coordinates	Nucleotide change	AA change	Hom/Hct/Hem	References
The patient	CBS	Exon8	chr21:44485352	c.697 T > G	p.Tyr233Asp	Hct	–
	CBS	Exon6	chr21:44485755	c.502G > A	p.Val168Met	Hct	16
The parents	Mother of the patient					c.697 T > G	
	Father of the patient					c.502G > A

Therefore, the boy was diagnosed with the CBS deficiency and treated with a supplement of pyridoxine 50 mg daily, betaine 3.0 g bid and a low-Met diet in another children’s hospital. The ocular surgery was delayed because of the high risk of thrombosis.

At the three-month follow-up, the plasma homocysteine (Hcy) concentration was lowered to 75.0 μmol/L. The lens located in the inferior vitreous cavity while the pupils were mildly dilated in both eyes with normal intraocular pressure (15 mmHg in the right eye, 14 mmHg in the left eye). The best-corrected vision was 6/9.5 in the right eye (+ 12.00/− 2.00 × 80, SE + 11.00D), 6/24 in the left eye (+ 11.00/− 1.50 × 100, SE + 10.25D).

Pyridoxine, betaine as well as diet restriction was continued to reach the optimal target for the plasma Hcy at < 50 μmol/L [1]. To prevent pupillary-block glaucoma, mydriasis with tropicamide eye drops was suggested. The further plan included pars plana lensectomy combined with posterior chamber scleral fixation of an intraocular lens after the plasma Hcy was well controlled at the normal level. Low molecular weight heparin anticoagulation would be required preoperatively.

## Discussion

Homocysteine (Hcy) is formed in the catabolic pathway for methionine (Met), an essential amino acid taken up from animal protein digestion [5]. CBS deficiency impairs the conversion of Hcy to cystathionine and leads to Hcy and Met accumulation. The pathophysiology of CBS deficiency is not fully understood. It is suggested that elevated Hcy concentrations modify sulfhydryl groups on proteins and interfere with the cross-linking of sulfhydryl groups in proteins such as elastin, which is considered to lead to the ectopia lentis and skeletal abnormalities [4]. Elevated Hcy concentrations also alter intracellular signaling and cause endoplasmic reticulum stress, which can result in thromboembolism and vascular disease [6, 7].

A low-Met diet and supplement of pyridoxine (vitamin B_6_) were prescribed to lower the Hcy concentration as close to the normal level as possible. Early-diagnosed patients have opportunities to maintain normal growth and to avoid severe complications [8, 9]. For late-diagnosed patients, reasonable control of serum homocysteine level can reduce the risk of further complications, such as seizures, abnormal behaviors, and thromboembolic disease [10–12].

However, it is not every patient that is sensitive to the supplement of pyridoxine. It depends on the location of mutations on the CBS gene. To date, over 900 CBS alleles in patients of varied ethnic origins were characterized and 164 different pathogenic genetic variants were observed [13]. Only a few mutations present in the homozygous state were shown to have genotype/phenotype correlations, concordance between pyridoxine responsiveness and clinical phenotype.

Some specific mutations cause a severe pyridoxine non-responsive form of the disease when inherited in the homozygous state, such as the c.919G > A (p.G 307S) tested in Ireland, the c.572C > T (p.T191M) in Spain, Portugal and South America [14, 15]. On the contrary, the c.833 T > C (p.I278T) variant, which was derived from Europeans, manifested a mild pyridoxine-responsive type of CBS deficiency when homozygous [13]. Compound heterozygotes carrying the c.833 T > C (p. I278T) variant on one allele are partially pyridoxine-responsive in some patients, whereas totally not responsive in others [14, 15]. Therefore, such correlations are difficult to infer in individuals who are compound heterozygotes.

In this case of CBS deficiency, the CBS gene mutated at two different positions, NM_000071.2(CBS): c. 502G > A, and NM_000071.2(CBS): c.697 T > G. The missense mutation c.502G > A was reported as a pathogenic mutation [16]. But paternal manifestation was clinically negative. This was probably due to heterozygosity at the CBS gene. For c. 697 T > G (p. W233N) in the proband, which was inherited from his asymptomatic mother, there was no previous literature report. It was confirmed by Sanger sequencing of samples from the patient and his mother. The nearby missense variants (p. A231P, p. H232D, p. D234N, p. E239K) have been reported in the Human Gene Mutation Database in association with homocystinuria, supporting the functional importance of this region of the protein [17]. The result of Condel, SIFT and PolyPhen also supported that the missense single nucleotide variant was deleterious for protein activity [18–20].

We found that our patient was a compound heterozygote at c. 502G > A and c. 697 T > G. Fortunately, after 3 months of diet control of Met and supplement of pyridoxine, his plasma Hcy concentration was lowered to 75.0 μmol/L, which met the criterion for metabolic control [1, 3]. The result indicated that this compound heterozygote was pyridoxine-responsive.

In this case, the ectopia lentis significantly manifested as the lens moved back and forth between the inferior vitreous cavity and the anterior chamber. The recurrent movements of the lens apparently induced mechanical injury to the iris, resulting in iris atrophy.

The blockage of aqueous humor due to dislocated lens in the anterior chamber led to the acute elevation of intraocular pressure when the pupil was not large enough. Furthermore, the frequent changes of lens position caused significant uncorrected refractive fluctuations, which happened from a very young age resulting in amblyopia. When the lens dislocated into the inferior vitreous cavity, the refraction was high hyperopia due to the absence of lens refractive power. However, it changed into severe myopia when the lens moved forwards into the anterior chamber, thus moving the focal point in front of the retina significantly. Frame glasses could not precisely correct such dramatically changing refractive errors. Thus, amblyopia could be developed due to the refractive fluctuations [21].

The most effective treatment might be surgically removing the lens with intraocular lens implantation [21–25]; therefore, the postoperative refraction could facilitate amblyopia treatment. Meanwhile, the replacement of the recurrent dislocated lens with an IOL might effectively eliminate the recurrence of pupillary block glaucoma. However, the parents could not bear the risk of thrombosis as a complication of surgery. Therefore, we arranged a reasonable medical treatment, including mydriatic and anti-glaucoma eye drops; and the intraocular pressure was carefully monitored at each follow-up. If the boy accepts lensectomy with IOL implantation, the visual acuity may improve gradually to a satisfactory level; as a teenager, he still has a potential to acquire better vision with reasonable amblyopia treatments.

Although patients with Marfan’s syndrome usually demonstrate a tall and thin appearance with arachnodactyly, the direction of partial lens dislocation is commonly supertemporal, which may be detected as the upper dislocation of the lens with the margin being observed at the pupil through the slit lamp examination [26]. However, the typical ocular manifestation of the CBS deficiency is a total lens dislocation in the anterior chamber, or sinking into the lower part of the vitreous cavity [1]. If a child is referred to an ophthalmologist with lens dislocation, the serum homocysteine concentration should be tested when the traumatic injury is excluded. Since CBS deficiency is an autosomal recessive condition, family members at risk for the disease should be tested by measuring Hcy or, in exceptional cases, by molecular genetic or enzymatic analysis.

In this case, the metabolic control did not affect the eye structure nor the refraction. The dislocated lens did not change due to the rupture of most of the ciliary zonules. However, for early diagnosed patients without complications, the metabolic control treatment can realistically aim to prevent all the complications of CBS deficiency including ectopia lentis, whilst maintaining normal growth and nutrition [1]. An enzyme replacement therapy in the mice model successfully demonstrated that the early control (initiated 3 weeks of age and continued for a further 9 months) of plasma Hcy prevented the rupture of the ciliary zonules, thus rescued the ocular phenotype [27]. For late-diagnosed patients, the aim is to prevent further complications, especially thromboembolic disease. Raised Hcy concentrations cause endothelial dysfunction as well as impaired thrombolysis, which may be responsible for thromboembolism and vascular disease. Sound control of plasma Hcy is effective to lower the risk of thrombosis and other complications.

Dehydration and infection increase the risk of venous thrombosis, particularly in children [28, 29]. It is important to ensure patients with CBS deficiency are well-hydrated at all times, especially when sick and during anesthesia and surgery.

Monitoring of plasma Hcy, AA, folate and vitamin B12 is recommended in all patients [1]. Patients on dietary treatment require regular nutritional assessment and additional tests to avoid poor growth or malnutrition. Restricting intake of Met reduces Hcy production. However, children with severe CBS deficiency still require a low-Met intake to maintain normal growth [30].

Bone density scans (DEXA) should be tested every 3–5 years from adolescence [31]. Vitamin D and calcium supplements should be prescribed if osteoporosis exists.

For long-term uptake of pyridoxine and betaine, adverse effects such as peripheral neuropathy should be monitored in the regular follow-up [32].

As cystathionine beta-synthase resides in the liver, liver transplant has been performed and manifested as an effective treatment. Some cases of liver transplant were reported with successful metabolic control without any dietary restrictions [33–35]. Meanwhile, research indicates that liver-targeted gene therapy has effectively lowered the concentration of Hcy in a CBS-deficiency mouse model, bringing hope for the possible treatment of the disease [36, 37].

## Conclusion

Children with CBS deficiency may suffer from ectopia lentis, glaucoma and/or amblyopia. The gene detection in this case showed a mutation at c.697 T > G, which was never reported before. The patient was pyridoxine responsive and well controlled by medicine.

## Data Availability

The genetic datasets generated and/or analysed during the current study are available in the CNGB Sequence Archive of China National GeneBank DataBase repository, accession number CNP0001806.
